# Botulinum Toxin Use in the Lower Urinary Tract

**DOI:** 10.1100/tsw.2007.150

**Published:** 2007-03-30

**Authors:** Shelby N. Morrisroe, Michael B. Chancellor

**Affiliations:** Department of Urology, University of Pittsburgh School of Medicine, Pittsburgh, PA, USA

**Keywords:** botulinum toxin, incontinence, neurogenic bladder

## Abstract

Botulinum toxins are well known for their ability to disrupt neurotransmission and cause muscle paralysis. Recently, urologists have discovered their beneficial effects in patients with neurogenic and overactive bladder conditions. This review is intended to provide a quick overview for urologists of the structure, function, and clinical uses of botulinum neurotoxin A in the lower urinary tract.

## INTRODUCTION

Neurogenic detrusor overactivity (NDO) and idiopathic detrusor overactivity (IDO) are commonly occurring urologic conditions caused by involuntary contractions of the detrusor muscle. Anticholinergic medicine is considered the "gold standard" for treatment of such lower urinary tract disorders. However, the not insignificant population of patients who fail this conservative measure for lack of efficacy or intolerable side effects has led to the investigation of alternative treatments. One such targeted therapy that is gaining increasing interest is intradetrusor injections of botulinum toxin, most commonly botulinum neurotoxin type A (BoNT/A). While high doses can be lethal to humans, very low dosing and targeted administration methods have harnessed these toxins for therapeutic uses.

Since the use of BoNT/A in the bladder was first described by Dykstra and colleagues in 1990[1], its clinical applications in urology have been expanding at a rapid rate. Current uses for BoNT/A in the lower urinary tract include treatment of NDO, IDO, detrusor external sphincter dyssynergia (DESD), and more recently, benign prostatic hyperplasia (BPH). Studies have shown BoNT/A to be of benefit clinically in DO, as demonstrated by improvement in incontinence scores, urodynamic parameters, as well as in quality of life (QOL) assessments[2,3]. Clinical data also suggest that there are similar responses to BoNT/A for patients with neurogenic and idiopathic causes of incontinence[4]. However, a definitive explanation of how BoNT/A exerts its effect on the bladder is still unknown. A step-wise mechanism of action for the botulinum toxins was first suggested by Simpson in 1979[5] and involves inhibition of acetylcholine (ACh) release at the presynaptic cholinergic neuromuscular junction (NMJ), with resultant inhibition of detrusor muscle contraction. More recently, botulinum toxins have been shown to inhibit other vesicle-bound neurotransmitters in afferent and efferent pathways of bladder wall and urothelium[6,7]. BoNT/A has received regulatory approval for a number of conditions characterized by excessive striated muscle contractility or tonus (e.g., cervical dystonia, blepharospasm, strabismus). Although its effects in smooth muscle appear to be similar to those in striated muscle, it is not approved by the Food and Drug Administration for use in the bladder (currently in Phase III FDA trial). However, as the potential uses of BoNT/A continue to expand within the field of urology, it is important to be familiar with the mechanism by which the toxin works and how it may be applied clinically.

## BOTULINUM NEUROTOXIN TYPE A: INFLUENCE OF STRUCTURE ON FUNCTION

Botulinum toxins are produced by the anaerobic, gram-negative organism *Clostridium botulinum*. There are seven immunologically distinct strains (serotypes A through G), although only two (types A and B) are commercially available. Type A is the most prolonged and has been the most extensively studied. BoNT/A is initially synthesized as a single, inactive chain of 1285 amino acids with a molecular weight of 150 kDa[8]. After activation by endogenous proteases, the resulting dichain polypeptide contains a heavy chain connected by a disulfide bond to a light chain and is folded into three functional domains that directly guide the toxin's action on target cells[9,10,11].

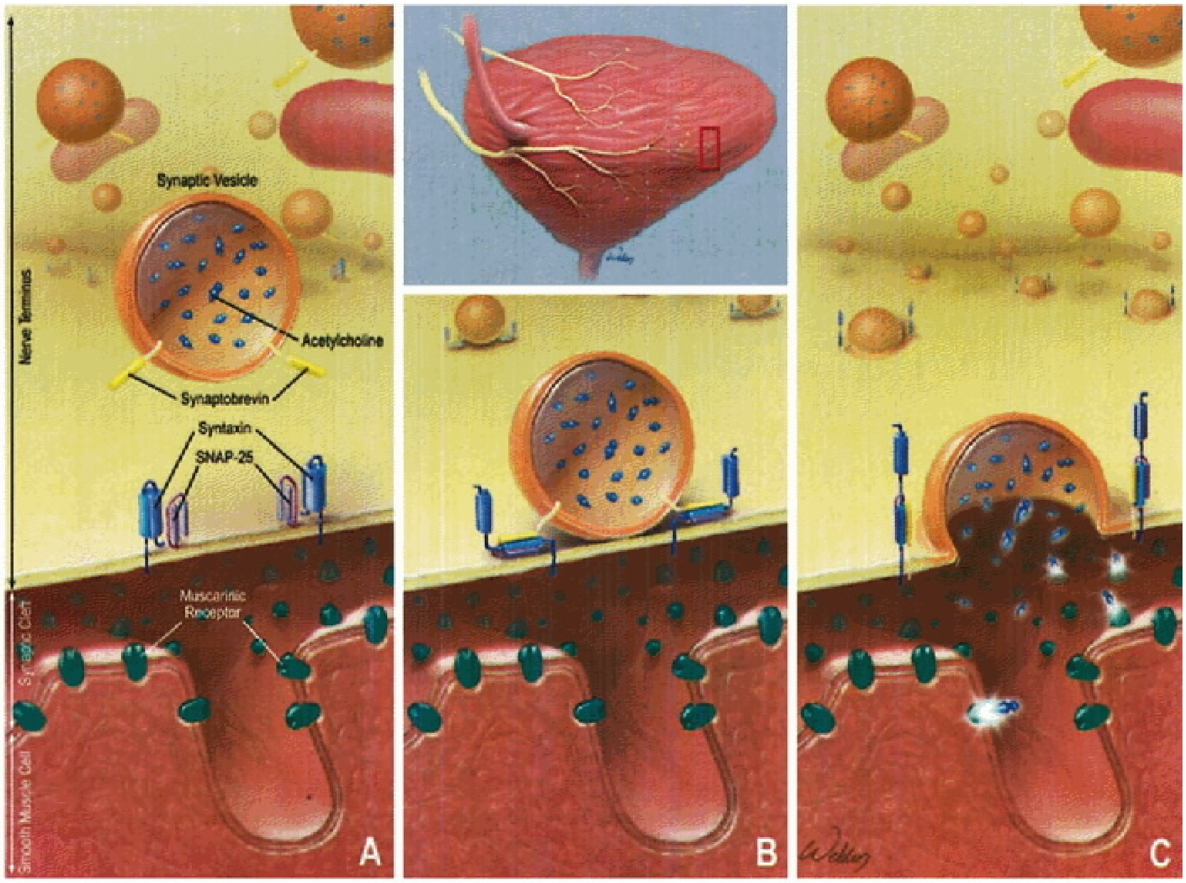

**FIGURE 1.** Normal fusion and release of ACh from nerve terminals via interaction of vesicle and membrane bound (SNARE) proteins. (A) Parasympathetic nerves innervating bladder with nerve terminal in unactivated state displaying numerous vesicles containing neurotransmitter ACh. SNAP-25, synaptosomal associated membrane protein. (B) Following nerve activation, assembly of SNARE protein complex (e.g., synaptobrevin, SNAP-25, and syntaxin) occurs, which leads to (C) release of ACh and activation of postjunctional muscarinic receptors, resulting in bladder contraction.

### Action at the Neuromuscular Junction

Under normal physiological conditions, action potentials within motor neurons stimulate the release of ACh into the synaptic cleft. Resultant elevations in $Ca^{2+}$ concentration cause ACh-containing synaptic vesicles to fuse with the presynaptic membrane where they release the neurotransmitter into the NMJ. Liberated ACh then diffuses across the synaptic cleft and binds to receptors on the surface of muscle cells, triggering muscle contraction (Fig. 1).

Injection of BoNT/A causes inhibition of this signal transmission at the NMJ in four discrete stages: binding, internalization, translocation, and exocytosis inhibition. After binding to the nerve terminal via its heavy chain binding domain, a receptor-mediated endocytotic process occurs[12,13,14,15]. The heavy and light chains then dissociate to increase the toxin's hydrophobicity, although only the light chain undergoes translocation into the cytosol[16,17,18]. The light chain then releases proteolytic fragments into the cytosol that target various vesicle and target membrane proteins including the translocation protein synaptosomal associated membrane protein (SNAP-25). ACh vesicle exocytosis and the resultant muscle contraction are therefore prevented (Fig. 2).

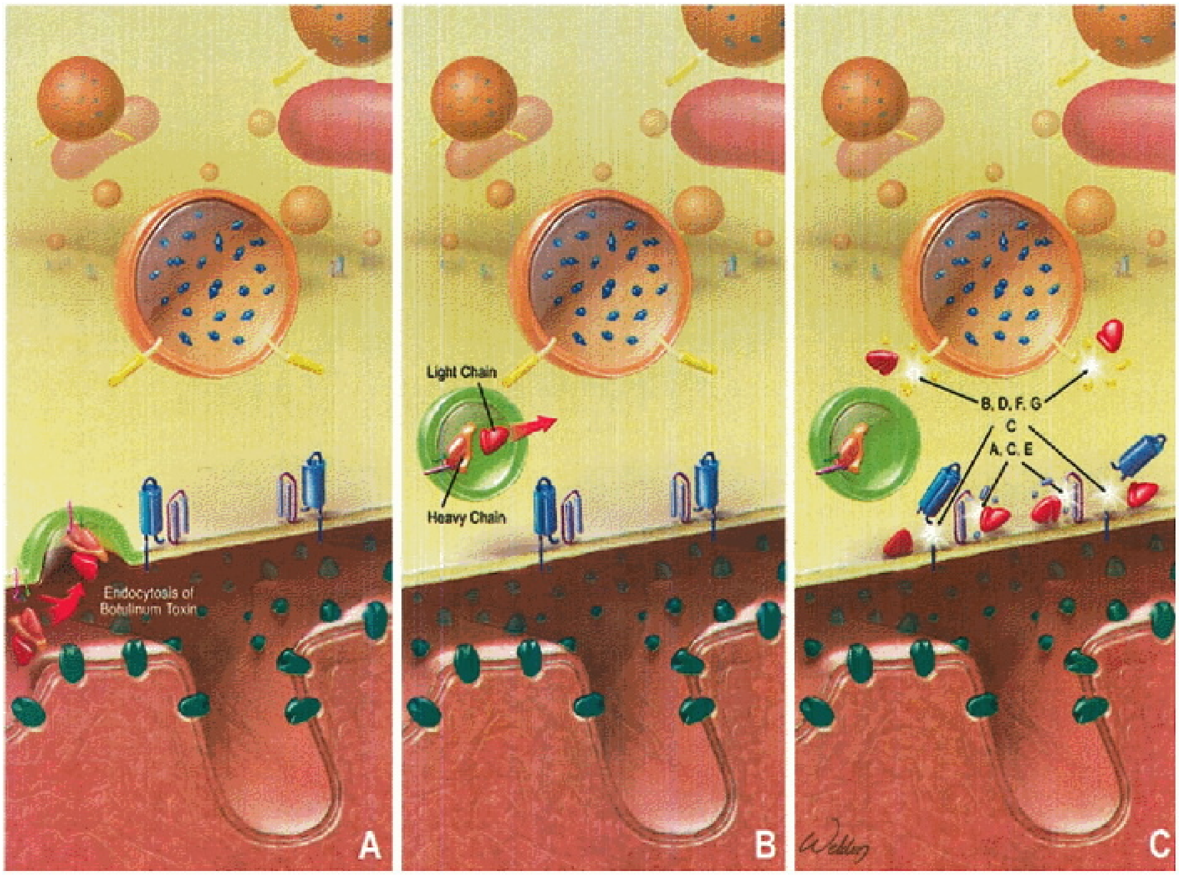

**FIGURE 2.** Parasympathetic nerve terminal. (A) Binding of toxin heavy chain to surface receptor and internalization of toxin within nerve terminal. (B) Translocation of light chain into cytosol. (C) Inhibition of neurotransmitter release by cleavage of specific SNARE proteins. *A* to *G*, botulinum toxin serotypes.

None of the Clostridial neurotoxins cause death of neurons. Rather, they temporarily inactivate transmission at the NMJ causing compensatory nerve sprouting, more prominent in striated muscle cells[19,20,21,22]. The effects are only temporary and studies have shown that BoNT/A produces no persistent changes in muscle fiber internal architecture after recovery from paralysis[23]. When exocytosis at the parent terminal eventually recovers, the sprouts are retracted and nerve terminal functioning returns to normal[22,24].

### Effect on the Detrusor Muscle

Although the therapeutic efficacy of BoNT/A in the lower urinary tract suggests that its effects on smooth muscle are similar to those on striated muscle, more detailed information is needed to establish the exact mechanism and site of action of the toxin in the bladder. Along with ACh, the neurotransmitter ATP has been implicated in the generation of unstable contractions in both spinal cord injury (SCI) and DO[25,26,27,28,29]. Studies on guinea pig and rat bladder strips have shown that BoNT/A is capable of inhibiting release of both ACh and ATP, providing further support for its use in treating patients with these disorders[30,31,32].

Haferkamp et al.[33] looked at ultrastructural changes in overactive human detrusor tissue following BoNT/A injection. Twenty-four patients with NDO collectively had 30 biopsies taken before and 3 months after BoNT/A injection, and during the wearing-off phase of the toxin’s efficacy. No significant changes were observed in muscle cell fascicles, intercellular collagen content, or muscle cell degeneration when comparing biopsies taken before and after BoNT/A administration, although these results cannot be extrapolated to the possible structural effects of repeat injections.

### Effect on Sensory Pathways

Aside from temporary chemodenervation of skeletal or smooth muscle, BoNT/A injection may have an antinociceptive effect on both acute and chronic inflammatory pain. This effect was originally suspected based on decreases in pain associated with conditions such as migraine headaches, chronic myofascial pain, and spasmodic torticollis[34,35,36]. Recent studies show that inhibition of neurotransmitter release occurs not only from efferent nerve endings, but from sensory nerve terminals and/or urothelium as well[37], further supporting the role of BoNT/A as an antinociceptive agent. The bladder urothelium may play an important role in the sensory transduction mechanisms modulating micturition, particularly in conditions of increased sensory nerve transmission following chronic inflammation and SCI[32,33].

Sensory axons in the bladder contain both calcitonin gene-related peptide (CGRP) and substance P (SP). CGRP and SP function as inflammatory response mediators and are released by primary afferent sensory fibers in response to noxious stimuli, causing greater excitability of nociceptive neurons[38,39]. Intravesical administration of BoNT/A blocks acetic acid–induced bladder pain responses and inhibits CGRP and SP release from afferent nerve terminals[39,40], supporting the clinical application of BoNT/A for the treatment of disorders such as interstitial cystitis and sensory urgency[41].

## FROM CELLULAR ACTION TO CLINICAL EFFECT

Therapy with BoNT/A would appear to not only help alleviate inappropriate detrusor muscle contractions in conditions such as SCI, NDO, and IDO, but in view of its proposed nociceptive properties, it could also provide substantial relief of hyperalgesia associated with chronic inflammatory states, sensory urgency, and interstitial cystitis. Selective injection permits specific paralysis of the detrusor muscle and modulation of sensory mechanisms, reducing episodes of incontinence and urgency without producing bladder neuromuscular paralysis and urinary retention.

## Neurogenic Detrusor Overactivity

Schurch and colleagues[42] performed a placebo-controlled, prospective, randomized study of the efficacy of BoNT/A intradetrusor injections in patients with NDO caused by SCI or multiple sclerosis. Fifty-three patients were randomized to receive a single dose into the detrusor of BoNT/A (200 or 300 U) or placebo, and followed for 24 weeks. BoNT/A was injected in 30 sites in the lateral walls of the bladder. Voiding diaries and urodynamic parameters (maximum cystometric capacity, reflex detrusor volume, and maximum detrusor pressure during bladder contraction) were used to provide objective measures of the treatment effect on bladder function while QOL questionnaires were used to assess subjective improvement. Their results showed post-treatment decreases in incontinence episodes of approximately 50% from baseline in the two BoNT/A groups, but not in the placebo group. Both BoNT/A groups also showed significant improvements in bladder function as assessed by urodynamics and in patient QOL. Benefits were sustained through the 24-week follow-up, demonstrating that a single treatment of intradetrusor injection of 200 or 300 U BoNT/A is effective for improving incontinence, bladder function, and QOL.

## Idiopathic Detrusor Overactivity

One prospective, nonrandomized, ongoing study by Schmid et al.[2] evaluated the efficacy and safety of intradetrusor injections of BoNT/A in patients with IDO. Twenty-three men and 27 women with IDO, including urgency-frequency syndrome, and incontinence despite the administration of maximal doses of anticholinergics were consecutively treated with injections of 100 U BoNT/A in the detrusor muscle at 30 sites under cystoscopic guidance. Clinical, urodynamic, and QOL assessments were performed at baseline, and 4, 12, and 36 weeks after BoNT/A treatment. After 4 and 12 weeks, 88% of patients showed significant improvement in bladder function in regard to subjective symptoms, QOL, and urodynamic parameters. Urgency disappeared in 82% of the patients and incontinence resolved in 86% within 1 to 2 weeks after treatment. Mean frequency decreased from 14 to 7 micturitions daily and nocturia decreased from 4 to 1.5 episodes per night. Mean maximal bladder capacity increased 56% from 246 to 381 ml and mean detrusor compliance increased from 24 to 41 ml/cm $H_2O$. There were no significant side effects except temporary urine retention in four cases. Mean efficacy duration ± SD was at least approximately 6 ± 2 months. These results show that intradetrusor BoNT/A injections may be an efficient and safe treatment option in patients with severe overactive bladder resistant to conventional treatments.

Werner and colleagues[43] also demonstrated that BoNT/A is an effective and safe treatment option for patients with IDO incontinence. Their prospective, nonrandomized study included 26 women who were injected with 100 U BoNT/A at 30 sites in the detrusor muscle. Results showed that 14 were dry after 4 weeks. With continued follow-up, 13 of 20 were found to be dry after 12 weeks, and 3 of 5 were dry after 36 weeks. Only two women overall failed to respond and the only reported complication was urinary tract infection in nine. BoNT/A injection at a dose of 200 U was also found to be effective as a suburothelial injection in a study by Kuo[44]. Of the 20 patients studied, 11 were men with benign prostatic hyperplasia after transurethral prostate resection, and nine were women with IDO. Their results of treatment with this dose showed significant improvement in IDO, but also demonstrated some impairment of bladder sensation and voiding efficiency. After 3 months of treatment, nine patients regained continence, eight showed improvement, while treatment failed in only three. Urinary hesitancy resulted in 15, transient urinary retention in six, urinary tract infection in seven, and hematuria in one.

## Detrusor External Sphincter Dyssynergia

Smith et al.[3] reported their results of urethral sphincter BoNT/A injection in 68 patients predominantly with multiple sclerosis who had DESD and/or were physically unable to perform intermittent catheterization. 100 to 200 U BoNT/A were combined with 4 ml sterile saline and injected into the external urethral sphincter using a rigid cystoscope and a cystoscopic collagen injection needle. Approximately equal aliquots of BoNT/A were injected into the four quadrants of the urethral sphincter at the 3-, 6-, 9-, and 12-o'clock positions. Alternatively, a fine-gauge spinal needle was used in some female patients to inject BoNT/A periurethrally. Injections were directed deeper than collagen injections to target the nerve terminals innervating the skeletal muscle. An additional 0.3 ml of normal saline was injected at the end of the procedures to ensure that the remaining toxin left in the needle dead space was delivered. Results showed an improvement in mean postvoid residual from 88 to 240 ml. The mean cystometric capacity improved from 198 to 241 ml. There was also a decrease in maximal voiding pressures from 81 to 52 cm $H_2O$. The incidence of retention requiring catheterization decreased by 80%, and patients reported decreased infection rates coincident with improved neurologic functioning (i.e., decreased spasticity and fatigue). Three patients did develop new stress urinary incontinence or a worsening of existing incontinence after treatment despite improvement of other parameters. Urethral injections improved the patients' ability to empty their bladders more effectively, as evidenced by the decrease in the mean PVR urine volume, and improved their overall medical well-being, as evidenced by the decreased infection rates and improvement in neurologic status (i.e., decreased spasticity and fatigue). In addition, repeated injections usually lasted longer than the first injection, with some patients maintaining efficacy for more than 1 year.

## Injection Technique

Smith et al. also describe their technique for detrusor muscle injection[3,45], choosing to inject 10 to 40 spots (i.e., depending on the dosage of toxin used and the condition treated — neurogenic vs. idiopathic bladder dysfunction [Fig. 3]). In addition to injections in the lateral walls of the bladder, the base and trigone areas are also targeted. This allows injection into the bladder's dense sensory innervation, specifically C-fibers, in the trigone and bladder neck, maximizing results. This strategy may be important particularly in the context of BoNT/A's potential antinociceptive effects. Some investigators avoid trigonal injection due to the theoretical concern for the development of vesicoureteral reflux. Smith et al. report that although they have not formally analyzed patients for postinjection vesicoureteral reflux, no clinical episodes of pyelonephritis have been observed. The authors do, however, recommend avoiding the bladder dome and posterior walls to prevent inadvertent perforation and bowel injection. Table 1 compares the injection technique of these authors and others in both the adult and pediatric populations.

**TABLE 1**

| Study | Indication | Injection Sites | No. of Injections | BoNT/A | Saline |
|---|---|---|---|---|---|
| Smith et al.[3] | NDO | Bladder lateral walls, trigone, base (excluding posterior wall and dome) | 30–40 | 200–300 U | 20–30 ml |
| | DESD | External urethral sphincter | 4 | 100–200 U | 4 ml |
| Smith et al.[45] | IDO, interstitial cystitis | Bladder trigone, base | 10 | 100 U | 10 ml |
| Schmid et al.[2] | IDO | Bladder lateral walls (excluding trigone) | 30 | 100 U | 10 ml |
| Schurch et al.[42] | NDO | Bladder lateral walls | 20–30 | 200–300 U | 20–30 ml |
| Altaweel et al.[47] | Pediatric NDO | Bladder lateral walls (excluding trigone, bladder neck) | 10 U/site | 5 U/kg (max 300) | 1 ml/10 U |

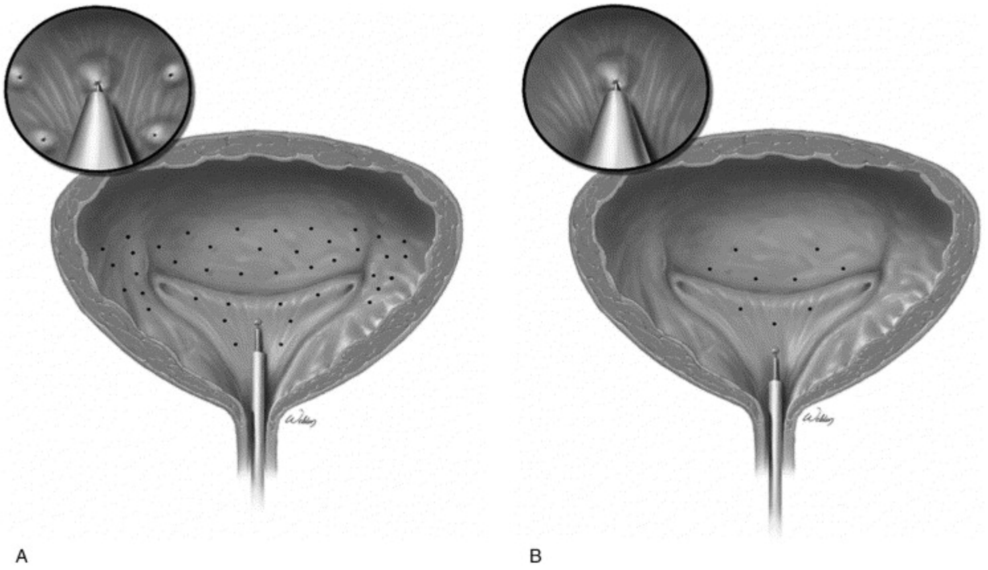

**FIGURE 3.** Bladder injection technique. (A) Depiction of approximately 30 submucosal injections throughout trigone, base, and lateral walls of bladder used in neurogenic detrusor hyperactivity. (B) Depiction of modified injection technique in IDO and IC populations in which ten submucosal injections are administered at bladder trigone and base, targeting sensory nerve pathways and limiting toxin dosage and distribution.

Accumulating data indicate that BoNT/A is an effective and well-tolerated treatment of urinary dysfunction in patients with NDO, IDO, DESD, and sensory disorders in whom conservative treatment has failed[46]. In contrast to the daily oral intake of anticholinergics, BoNT/A injections have a long-lasting effect up to 9 months, and patients typically experience little or no adverse effects. Further, effectiveness of treatment does not seem to be markedly affected by the large range of BoNT/A doses used in clinical studies. BoNT/A injection therapy also appears to have similar benefits in the pediatric population[47]. It may even reduce the risk of vesicoureteric reflux and subsequent renal impairment in children with NDO, postponing or reducing the necessity for bladder augmentation surgery[47,48]. Relative contraindications of BoNT/A treatment include peripheral motor neuropathy (e.g., amyotrophic lateral sclerosis), NMJ disorders (e.g., myasthenia gravis or the Lambert-Eaton myasthenic syndrome), and concomitant treatment with aminoglycosides or other agents interfering with neuromuscular transmission.

There is yet to be a standardized dosage and treatment protocol and further studies are needed to determine the effect of dose, volume, and pattern of injection on efficacy, safety, and duration of action.

## BOTULINUM TOXIN DIFFERENTIATION

Currently, two types of botulinum neurotoxin are available in the U.S.: type A (Botox®, Allergan, Irvine, CA) and type B (Myobloc®, Elan Pharmaceuticals, Inc., San Francisco). Two other formulations of botulinum neurotoxin A are available in Europe (Dysport®, Ipsen Ltd. Slough, U.K. and Xeomin®, Merz Pharma GmbH Frankfurt am Main, Germany). Potency of the toxins is expressed in units of activity. One U of Botox® is approximately equivalent to 3–5 U Dysport®. The Botox® formulation is comprised of 900-kD neurotoxin complexes, whereas the Dysport® formulations is a mixture of 500- to 900-kD neurotoxin complexes.

BoNT/A, when used clinically, has by far the longest duration of activity, inducing muscle paralysis between 4 and 9 months. In contrast, the effects of BoNT/B and BoNT/F last only a matter of days, and in the case of BoNT/E, only a matter of hours[49].

Clinical observations report that Botox® is associated with a lower rate of adverse events than Dysport®[50,51]. These clinical reports are consistent with evidence that suggests that the larger, more uniform size of the Botox® neurotoxin complex protein (900 kD) minimizes unwanted migration that can lead to increased adverse events[52]. Thus, differences in average BoNT/A complex size between Botox® and Dysport® may, in part, explain differences in their respective adverse event profiles through the mechanism of toxin migration.

## Adverse Events

Adverse events secondary to treatment with BoNT/A have been minor and serious complications or fatalities have not been reported. Although rare, the most common systemic adverse event is upper extremity weakness[53]. The most localized adverse event includes stress urinary incontinence[54,55,56]. There remains a potential need for initiation of CIC in patients for transient urinary retention[44]. Urinary tract infection is also reported, but at the same frequency as with other cystoscopic procdures[57].

## CONCLUSION

BoNT/A is promising across a wide variety of urologic disorders, including neurogenic detrusor hyperreflexia, overactive bladder, and possibly pain syndromes. It does this by binding to the nerve endings of muscles and blocking the release of ACh, resulting in inhibition of muscle contraction or of sensory afferent firing. Many advances have been made in our understanding of how BoNT/A works at a molecular level, yet questions remain. Further research is needed to explore the toxin's long-term effects on smooth muscle cells as well as to determine the effect of dose, volume, and pattern of injection on efficacy, safety, and duration of action. Clinical trials seeking regulatory approval of BoNT/A in the urinary bladder are currently underway.
